# Strengthening Insights in Microbial Ecological Networks from Theory to Applications

**DOI:** 10.1128/mSystems.00124-19

**Published:** 2019-05-21

**Authors:** Xiaofei Lv, Kankan Zhao, Ran Xue, Yuanhui Liu, Jianming Xu, Bin Ma

**Affiliations:** a Department of Environmental Engineering, China Jiliang University, Hangzhou, China; b Institute of Soil and Water Resources and Environmental Science, College of Environmental and Resource Sciences, Zhejiang University, Hangzhou, China

**Keywords:** evaluation, inference, interpretation, microbial ecological network, microbial interactions, network science

## Abstract

Networks encode the interactions between the components in complex systems and play an essential role in understanding complex systems. Microbial ecological networks provide a system-level insight for comprehensively understanding complex microbial interactions, which play important roles in microbial community assembly. However, microbial ecological networks are in their infancy of both network inference and biological interpretation. In this perspective, we articulate the theory gaps and limitations in building and interpreting microbial ecological networks, emphasize developing tools for evaluating the predicted microbial interaction relationships, and predict the potential applications of microbial ecological networks in the long run.

## Abstract (precis)

Networks encode the interactions between the components in complex systems and play an essential role in understanding complex systems. Microbial ecological networks provide a system-level insight for comprehensively understanding complex microbial interactions, which play important roles in microbial community assembly.

## PERSPECTIVE

The term “complex system” represents a system whose collective behavior is difficult to derive from a knowledge of the system’s components, such as the cooperation between billions of individuals in human society (1), the links between countless webpages of the Internet (2), or the interactions between thousands of genes within cells (3). Given the important role complex systems play in every aspect of the world, it is one of the major scientific challenges in understanding, describing, predicting, and controlling complex systems (4). Network science emerging from the dawn of the 21st century has been resolving this challenge by constructing an intricate network to encode the interactions between a system’s components. For instance, social networks determine the spread of knowledge, behaviors, and resources, communication networks are at the heart of modern communication systems, and gene, protein, and metabolic networks are prerequisites of life. Accordingly, A.-L. Barabási, the bellwether of the network science, claimed that “we will never understand complex systems unless we develop a deep understanding of the networks behind them” (4).

Microbial communities are also complex systems. Microorganisms form complex ecological interactions, including win-win relationships such as mutual cross-feeding and cooperation interactions, win-lose relationships such as predator-prey and host-parasite interactions, and loss-loss relationship such as competitive exclusion interactions (5). These microbial interactions are known to be critical properties of microbial communities and play important roles in microbial community assembly. Reconstruction of microbial ecological networks representing these interactions can advance our understanding of the complex behaviors in microbial communities, predict the effects of perturbations on community dynamics, and help with the engineering of complex microbial communities (6).

**FIG 1:**
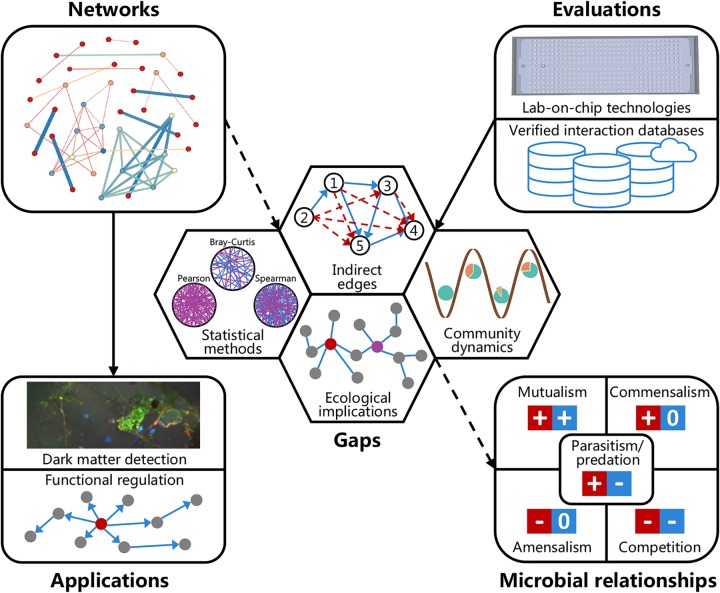
Microbial ecological networks are considered to represent the relationships of complex microbial communities. However, there are still some gaps in theory and limitations in network inference and interpretation, such as selecting the statistical method, eliminating indirect edges, describing ecological implications description, and reviving work on network evolution. Hence, we need to evaluate the predicted interaction relationships through developing tools like lab-on-chip technologies and public verified interaction databases. Ultimately, it could be used to detect microbial dark matter and regulate microbial community functions.

Although the potential value of inferring and interpreting microbial interaction networks has been known, inference and interpretation of microbial interaction networks are far from straightforward. In this perspective, we consider the gaps in theory and limitations in measuring microbial interactions, identifying indirect edges, understanding biological implications, and describing network evolution (Fig. 1). Additionally, we look ahead to the applications of microfluidics, high-throughput culturing methods, and verified interaction databases for evaluating the predicted microbial interaction relationships. Finally, we predict the potential applications of microbial ecological networks in detecting microbial dark matter and regulating microbial community functions.

## FILLING IN THE THEORY GAPS IN NETWORK INFERENCE AND INTERPRETATION

A network is a catalog of a system’s components often called nodes or vertices and the interactions between them, called links or edges. For microbial ecological networks, nodes represent microbial taxa or environmental factors, and edges represent potential microbial interactions inferred from statistically significant similarities. There are different toolkits and pipelines using a range of different statistical measure methods, including Pearson and Spearman correlation, inverse covariance, Bray-Curtis dissimilarity, and maximal information (5). Given that statistical measure methods differ in their strengths and weaknesses, it is not straightforward to determine the most appropriate model. The method chosen has a large impact on the association patterns in the resulting networks. Weiss et al. (7) found that networks inferred with different approaches shared less than a third of edges. Additionally, edge directions in microbial ecological networks have critical implications in understanding ecological processes. Although most of the methods based on cross-sectional data are unable to infer directed network, directed edges can be generated from time series data or Lotka-Volterra dynamics (6). Hence, criteria for statistical measure selection based on experimental designs and data set characteristics will increase accuracy and facilitate network interpretation. A set of simulated data sets with definite interaction relationships could be useful for evaluating network inferring methods (6).

The final inferred microbial networks generally contain spurious indirect edges. If two species cooccur with an unreported factor, such as undetected microbial species and abiotic drivers, they may acquire an indirect edge from covariance because they are both affected by the same factor. Although correlation-based tools have not removed indirect edges, two papers published in 2013 introduced two different methods for recognizing indirect edges in correlation-based networks. Feizi et al. (8) introduced a deconvolution algorithm for inferring direct effects from a correlation matrix containing both direct and indirect effects. At the same time, Barzel and Barabási (9) developed a method to silence indirect effects in correlation-based network. However, the performance of these two methods has not been evaluated for microbial ecological networks. Lima-Mendez et al. (10) proposed a method for detecting indirect edges by checking the association patterns in environmental triplet of taxon-environment networks. This method detects indirect edges with the assistance of environmental variables, but it is impossible to use all the environmental factors in the systems.

The edges in networks for social interactions, Internet links, and protein or gene interactions are defined definitely and clearly. Accordingly, the topological properties of these networks can be clearly interpreted. However, the implications of topological properties of microbial ecological networks, such as modularity, transitivity, and assortativity, are unclear because the edges of microbial ecological networks are defined ambiguously (6). Moreover, correlation-based microbial ecological networks cannot infer amensalism and commensalism and differentiate mutualism and competition.

In reality, microbial communities are dynamic; hence, microbial ecological networks are evolving during community assembly processes. Mathematically describing evolving networks allows us to address the impacts of various processes on network topology and evolution (4). Although time series data sets have been used for inferring networks, those studies highlighted determining the directions of edges, rather than determining the evolution of networks (11).

## EVALUATING THE PREDICTED INTERACTIONS

At present, one of the defects in most of the microbial interaction network studies is that the interaction relationships in the inferred microbial interaction network lack further evaluations. Coculture experiments could provide substantial evidence for evaluating the relationships in the microbial interaction networks. However, coculture experiments in petri dishes cannot represent the complexity of the microbial interaction networks. Lab-on-chip technologies might provide a solution for screening coculture features on a large scale. Moreover, given that more than 99% of microbial species in natural environments are uncultured and unknown to us, microbial dark matter will hamper our understanding of the microbial interaction networks. On one hand, advances in culture-independent methods, such as metagenomics and single-cell sequencing (12), will provide necessary information of microbial dark matter in interpreting and evaluating the interaction relationships in the microbial interaction network. On the other hand, emerging high-throughput culturing methods, such as *iChip* and culturomics (13), could greatly expand the numbers of culturable microbial strains to foster coculture evaluations.

It is almost impossible to experimentally evaluate all the microbial interaction relationships in an inferred microbial ecological network in a single study. Lima-Mendez et al. (10) evaluated the predicted interactions with a list of known symbiotic interactions *sensu lato* built through screening the literature by a panel of four experts. Public biological databases, which began in the early 1980s when DNA sequence data began to accumulate in the scientific literature, have been playing critical roles in promoting the rapid development of biology. The molecular life sciences have been increasingly driven by and reliant on these open-access public databases, such as the EMBL-EBI’s ENA, the NCBI’s GenBank, and NIG’s DDBJ. Accordingly, we suggest that creating a public database for archiving microbial interaction relationships could bridge the gaps between inferring microbial ecology networks and evaluations of the networks and advocate understanding microbial ecology networks.

## HARNESSING MICROBIAL FUNCTIONS WITH MICROBIAL ECOLOGICAL NETWORKS

Lacking interactions with cooccurring microorganisms from *in situ* environments is one of the reasons that most microbial strains failed to be cultured in labs. Identifying cooccurring species of interesting uncultured microbial species in microbial ecological networks might help to create the necessary growth environments for uncultured microbial species. Theoretically, uncultured microbial species have the chance to be cultured when they grow together with their mutualistic, syntrophic, or parasitic partners. For mutualistic relationship, either cultured microorganisms could increase the fitness of their uncultured mutualistic partner. For syntrophic relationship, only uncultured species that depend on the nutrients, growth factors, or substrates provided by the other cultured partners have the potential to be isolated. For parasitic relationship, a cultured microbial host could help to enrich uncultured parasites. Moreover, if a predation relationship is obligate, a cultured microbial prey could help to enrich uncultured predators as well. Other interaction relationships such as competition, amensalism, and commensalism do not have the facilities for promoting microbial dark matter detection.

The small-world property of network induces that any two members in microbial community could interact with each other through a few intermediaries. Liu et al. (14) investigated the controllability of networks and demonstrated that many real-world networks could be controlled through a small number of vertices. Regulating the functions of microbial communities is one of the core objects of microbial ecology. Given the critical roles of microbial interactions in microbial community assembly processes, realizing the controllability of microbial community functions needs to employ network controlling theory to detect key vertices to control. Many engineering microbial strains performed well in laboratory experiments but are difficult to apply in practical environments (15). Understanding the controllability of microbial ecological networks could support improving their performances in practical environments by manipulating interaction networks of microbial communities.

In conclusion, network analysis is a valuable approach for comprehensively understanding microbial community structure and functions. However, researchers in systems microbiology have a long way to go to catch up with the advanced developments in network science.
